# Alopecia Areata of the Nails: Diagnosis and Management

**DOI:** 10.3390/jcm13113292

**Published:** 2024-06-03

**Authors:** Christin Pelzer, Matilde Iorizzo

**Affiliations:** 1 Department of Dermatology, Venereology and Allergology, Kantonsspital St. Gallen, 9000 St. Gallen, Switzerland; christin.pelzer@kssg.ch; 2 Private Dermatology Practice, 6500 Bellinzona, Switzerland

**Keywords:** nail, alopecia areata, pitting, trachyonychia, red lunula, leukonychia, treatment, management

## Abstract

**Background:** Alopecia areata (AA) is a common form of non-scarring alopecia characterized by acute hair loss. Nail involvement, though not always present, can occur in AA patients. Nail changes are more frequent in severe forms of AA and in children. **Methods:** Literature related to nail changes in AA was comprehensively reviewed after a search on the PubMed database without time restrictions in order to identify common clinical presentations and associated factors to aid clinicians with the correct evaluation and management of these dystrophies. **Results:** Nail changes in AA include pitting, trachyonychia, leukonychia, red lunula, and miscellaneous alterations such as longitudinal ridging and brittle nails. Nail changes are usually asymptomatic but, nevertheless, sometimes cosmetically disfiguring and can be associated with a reduced quality of life and impaired daily activities. **Conclusions:** Nail changes in AA may precede or follow hair loss and can occur as an isolated finding. Diagnosis may require a biopsy for definitive identification. Spontaneous improvement is possible, particularly in children, and treatment is not always necessary. Further research is, however, needed to establish a consensus on treatment approaches according to age and severity.

## 1. Introduction

Alopecia areata (AA) is a common form of non-scarring alopecia characterized by acute hair loss in the absence of cutaneous inflammatory signs. The disease usually starts abruptly with one or multiple patches that enlarge in a centrifugal way. The entire scalp (AA totalis) and body (AA universalis) may be affected. This disease can occur in all ethnic groups, ages, and sexes, despite different prevalence rates [1]. Pediatric AA has been estimated to be present in around 20% of all AA patients, and more than 50% of patients present their first episode before the age of 20 [2]. The psychological impact of AA is very severe due to its acute onset, unpredictable course, and unpredictable prognosis due to the unknown etiology and the influence of several negative factors [3,4].

Nail involvement is one of these negative prognostic factors. Not only hair follicles may be under attack by the cytotoxic T cell-mediated inflammatory infiltrate, but also nail germinative cells, predominantly those of the proximal nail matrix, that are also an immune-protected site [5]. The inflammatory infiltrate is able to disrupt normal nail keratinization but not its mitotic activity since it has no scarring potential. The more severe the inflammation, the more severe the nail changes will be. Nails and hair share some immune system and anatomical features (the hair matrix is mirrored in the proximal nail matrix), but the pathogenesis of nail changes and genetic factors influencing immune responses are not fully understood and studied as profoundly as hair changes [5]. According to the literature, nail changes are present in 7–84% (on average, 22%) of AA patients, with a more frequent association with severe forms of AA like AA totalis or AA universalis [6,7,8,9], but they can also occur as an isolated finding without any hair involvement. As children tend to develop severe forms of AA more frequently than adults, nail changes are seen more commonly in this age group [10]. Males also seem to be more commonly affected by nail changes than females [6,7,8,9]. However, opposite statements have also been made [11]. Nail changes are usually asymptomatic but, nevertheless, sometimes cosmetically disfiguring and associated with a reduced quality of life, possibly owing to the greater visibility and, in some instances, the lack of the possibility to cover them with nail polish. About one-third of AA patients with nail changes are seeking help and treatment for their condition [12,13]. 

This review aims to summarize the literature related to nail changes associated with alopecia areata, trying to help clinicians with better evaluation and management of AA-related dystrophies. All the relevant English articles from the PubMed database have been evaluated by the authors, without time restrictions, according to the following keywords: “alopecia areata,” “nails,” “nail dystrophy,” “trachyonychia,” “pitting,” “red lunula,” “leukonychia,” and “treatment.” Additional research was derived from published articles, which were scrutinized to identify pertinent references overlooked during online inquiries.

## 2. Clinical Presentation

**Figure 1:**
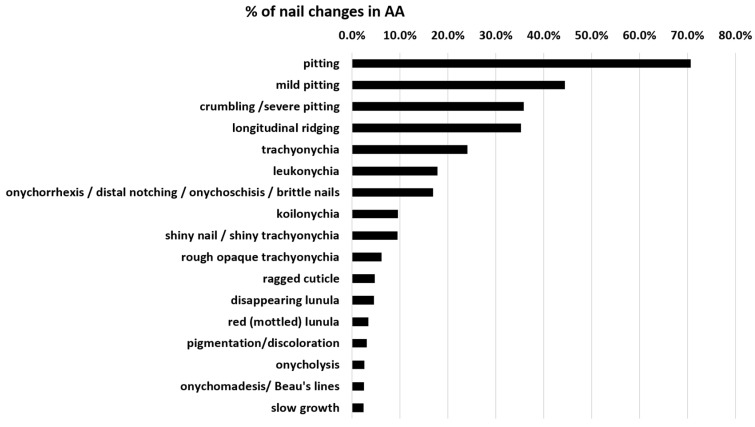
Distribution of specific nail changes in AA nails. Pitting is the most common, whereas a red-mottled lunula is rare but probably the most specific change that is associated with severe AA, as it almost exclusively occurs in AA universalis. Cumulative data were taken from Table 1 in the last column.

On average, nail alterations in AA are present in about 22% of patients. Specific nail changes are discussed below, and the frequency according to studies is displayed in Table 1. Figure 1 shows the percentage distribution of these AA nail dystrophies. Pitting, longitudinal ridging, trachyonychia, and leukonychia are the most common nail alterations. A red-mottled lunula is rare but seems to be associated with a more severe disease.

**Figure 2:**
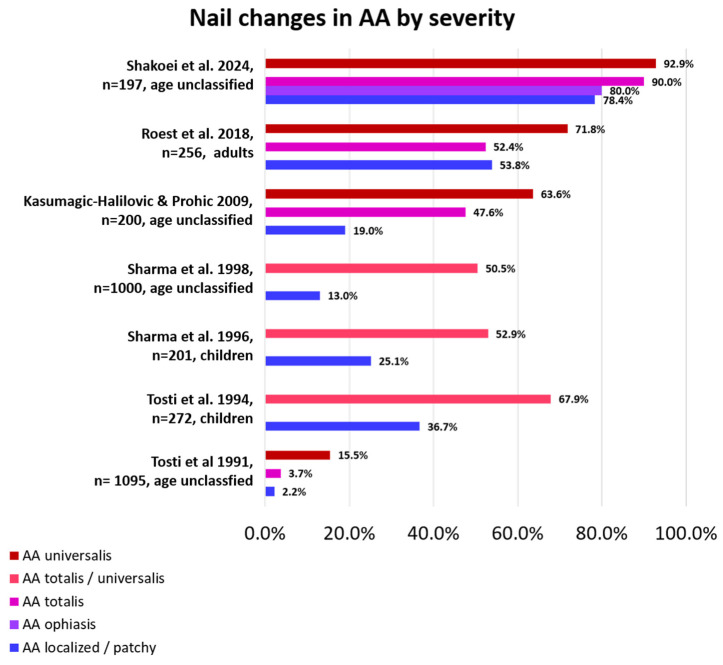
Percentage of nail changes according to AA severity by study. Nail changes occur more frequently in AA universalis than in less severely affected AA [6,7,8,10,12,14,22].

As stated above, nail involvement is not always present in patients with AA and is more frequently seen in severe AA than in mild AA (Figure 2). When present, nail changes may also go unnoticed, especially if they are subtle. Proper diagnosis and treatment of scalp and body hair loss are usually more important and pressing, so not every patient is bothered by nail abnormalities enough to see a physician, and many physicians do not take a look at the nails when focused on hair loss. Moreover, most of the time, nails improve in parallel with hair if the given treatment is successful, so baseline attention to them is scarce. The number of nails involved can vary from one to all, and disease severity may differ from nail to nail and from patient to patient, even if the hair loss is comparable among them. Depending on the anatomical area affected, different clinical signs can be evident and be present together in the same patient and even in the same nail [12,13]. Most of the changes are found within the proximal matrix, are less pronounced in the distal matrix, and are negligible in the nail bed. This means that most of the clinical signs of the nail are present in the superficial nail plate. In very limited situations, nails present cosmetic disfigurement, pain, and functional limitations.

### 2.1. Nail Pitting

**Figure 3:**
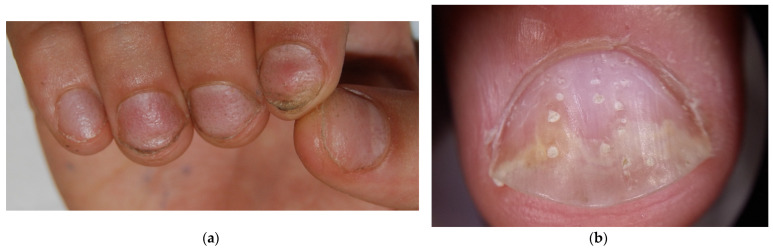
Comparison of nail pitting on AA nails (**a**) and nail psoriasis (**b**). Pits tend to be smaller, shallower, and more regularly distributed (grid-like pattern) on the surface of AA nails compared to those of psoriasis, where pits tend to be deeper, wider, and more randomly distributed.

The most common nail manifestation of AA is pitting. Nail pitting is punctate erosion (pits) in the nail plate surface commonly described in nail psoriasis, where they correspond to clusters of parakeratotic cells (nucleated and incompletely keratinized cells due to defective keratinization of the proximal nail matrix) easily detachable with minor trauma. Pits in psoriasis are typically deep and irregularly distributed, but they can also be more superficial. The depth and width of the pits relate to the extent of the matrix involved; their length is determined by the duration of the matrix damage. In AA, pits are very superficial and often go unnoticed—there is undulation or waving of the superficial nail plate rather than erosions and a grid-like geometric pattern [23] (Figure 3). Sometimes, they have been observed in a transverse line resembling Beau’s line, which represents a transversal depression or groove not involving the nail plate’s full thickness (in contrast to onychomadesis, which involves the full thickness of the nail plate) [10]. Pits are rarely observed in toenails. The same superficial and regular presentation of AA pitting can also be present in eczema or occupational nail dystrophy—the clinical diagnosis of AA pitting should then be performed with caution when only the nails are involved without the hair.

### 2.2. Trachyonychia

Another common nail manifestation of AA is trachyonychia. This word derives from the Greek word *trakos*, which means rough because the nails look like they are sandpapered. Trachyonychia can precede or follow the onset of AA by years and is more frequently observed in children (12% versus 3% of adults), where it also tends to affect all 20 nails [10]—this is the reason why this condition was formerly called “twenty nail dystrophy,” a synonym that is no longer appropriate and should be abandoned as the dystrophies do not always involve all 20 nails.

**Figure 4:**
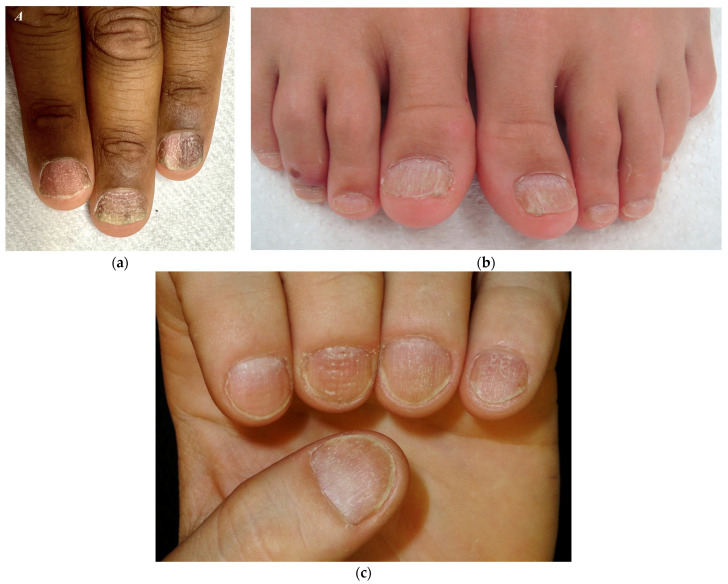
Trachyonychia—(**a**,**b**) Rough/opaque variant. Brittle and thin nails that appear lustreless; (**c**) Shiny variant. Multiple pits and superficial ridges while maintaining luster.

As reported by Robert Baran and co-authors, two forms can be distinguished: a rough/opaque and a shiny one [24] (Figure 4). The rough or opaque form is the most frequent and is due to severe and persistent inflammation of the proximal matrix, with the nail plate appearing lustreless with excessive superficial scaling, longitudinal ridging, and distal longitudinal splits. In contrast, the shiny form is due to a milder and intermittent inflammation that causes small punctate depressions distributed in a geometric order, reflecting the light and giving a shiny appearance to the nail plate [6,9,25,26,27]. The degree of inflammation may vary among the two subtypes. Patients with opaque trachyonychia are generally those who suffer more from nail fragility.

Trachyonychia, however, is not a distinctive disorder that allows the clinician to diagnose AA, but it is a presenting sign of several disorders that involve the nail matrix, like lichen planus, psoriasis, eczema, and many others [6,9,25,26,27,28,29]. Familial cases have also been described [6]. This is a very important consideration to take into account because the presence of more than one disease in the same patient has been reported [6,30,31]. Since the clinical appearance cannot safely distinguish between the abovementioned differential diagnoses, a biopsy is required to identify the causative disorder [6,32,33]. However, since trachyonychia is a benign condition with a positive prognosis, this procedure is not generally performed unless extremely necessary. 

### 2.3. Leukonychia

**Figure 5:**
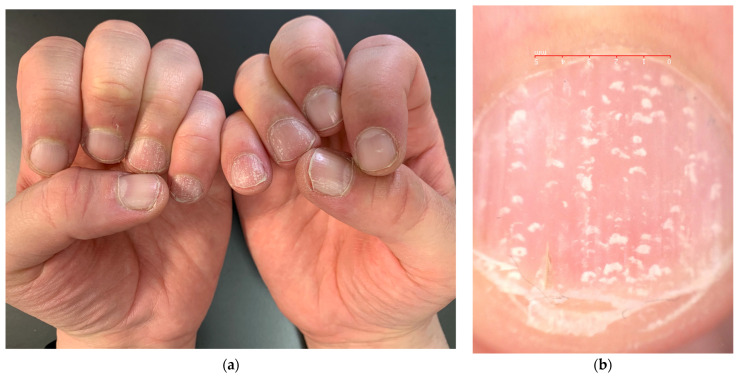
Punctate true leukonychia in a patient with AA—as pits, leukonychia spots are geometrically distributed ((**a**): clinical picture; (**b**): 10× magnification at dermoscopy).

Leukonychia means white nails. The term derives from the Greek word *leukós*, which means white. In AA patients, it usually presents in a punctate form (Figure 5), with spots geometrically distributed similar to pits. Leukonychia in AA is always true leukonychia, which means it is due to intrinsic matrix abnormalities: histologically odd-appearing parakeratosis with perinuclear vacuolization and keratohyaline granules are found in the intermediate and ventral nail plates. Keratin fibers have also been found dissociated, irregularly aligned, and fragmented on electron microscopy [34,35]. These focal abnormalities reflect visible light, preventing visualization of the underlying vascularized nail bed and making that area appear white. The abnormality, being located within the plate, cannot be modified until the plate grows out. Moreover, the white color does not change if pressure is applied. These characteristics make this type of leukonychia a true form [36]. The geometric spots of leukonychia, typical for AA, should be distinguished from those more randomly distributed and typically due to trauma that can be frequently encountered in the fingernails of mainly children but also adults [36].

### 2.4. Red Lunula 

**Figure 6:**
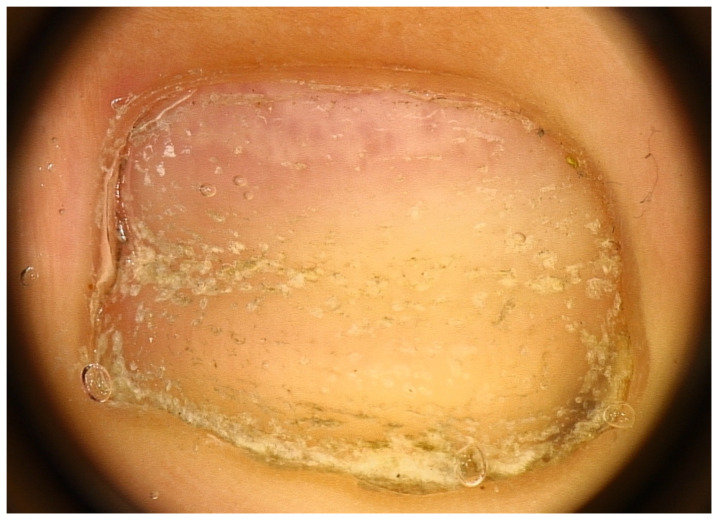
Red lunula—islands of red color resembling the color of the nail bed may appear in the lunula, giving rise to a motheaten appearance (mottled lunula).

Another nail change seen in AA nails is the red lunula. Note that the lunula (distal and visible part of the matrix) is most prominent on the thumb and great toe and may be partly or completely concealed by the proximal nail fold in other digits. The normal lunula has a white hue. In AA, islands of red color resembling the color of the nail bed may appear in the lunula, giving rise to a motheaten appearance (mottled lunula) with unaltered margins [37] (Figure 6). These red spots are attributed to dilated and tortuous vessels in the superficial papillary dermis of the distal matrix [38,39]—probably systemic or local factors are responsible for angiogenesis within the nail unit. The red lunula is not, however, a specific and diagnostic sign, as it is seen in many other diseases. At least for pediatric patients, literature reports red-mottled lunula only in association with pitting and AA universalis [10], sometimes even in association with Beau’s lines only [40]. In adult patients, an association with AA severity was also found, occurring almost exclusively in AA universalis [12,22,40].

### 2.5. Miscellaneous Nail Changes

Further nail changes, all unspecific, may include longitudinal ridging, onychorrhexis, Beau’s lines, onychomadesis, brittle nails, koilonychia, onycholysis, ragged cuticles, and brown discoloration [12,13,41,42]. The latter, however, has been reported in Indian children as well as Iranian patients with darker skin types [7,22], possibly being melanonychia due to activation of melanocytes related to the skin phototype and not necessarily to the inflammatory infiltrate itself. Secondary nail changes due to manipulation, like habit tic deformities, potentially caused by emotional stress, have also been reported in AA patients [43]. Onychomadesis and onycholysis are rare signs, but probably those impairing daily activities and causing pain the most. Especially in manually active patients, nails are required for proper dexterity.

## 3. Diagnosis

As stated above, it is not possible to make a diagnosis of AA in the nails just by looking at the clinical picture. When the hair is affected by AA and the nails present one of the abovementioned dystrophies, it can be assumed they are also affected by AA, even if this is not always true. Other inflammatory conditions, such as lichen planus, can, in fact, affect the nails in parallel with the AA of the hair [6,30,31], and if this needs to be ruled out, a biopsy is necessary, especially when the presenting sign is trachyonychia [44]. This is, however, not a routine procedure that can be reserved when there is no response to treatment and there is a need to know the type of inflammatory infiltrate to proceed further.

### 3.1. Biopsy 

**Figure 7:**
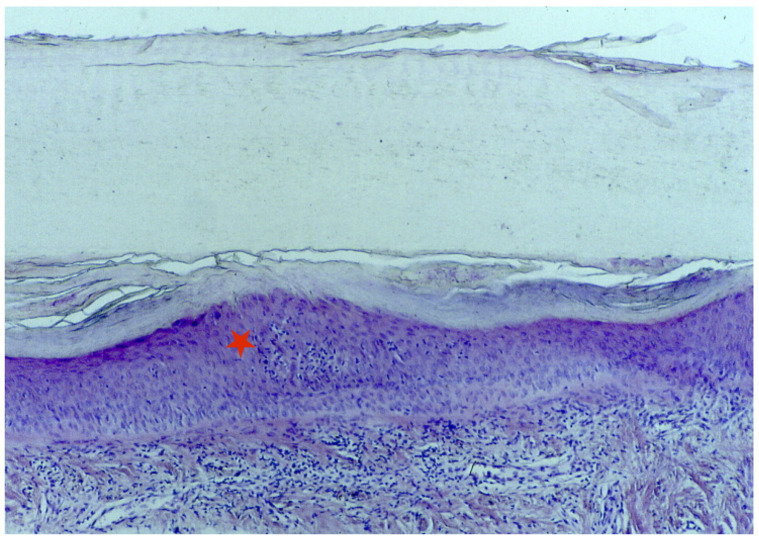
Histology of the AA of the nails shows spongiotic dermatitis in the matrix area (red star).

A longitudinal biopsy is preferred to a punch biopsy due to the larger amount of assessable tissue, preferably with the nail plate still attached. In the presence of trachyonychia, histology usually shows spongiotic dermatitis (Figure 7) with a dense lymphocytic epitheliotropic infiltrate in the proximal matrix, lymphocytic exocytosis into the nail epithelia, and often small spongiotic vesicles. These may be included in the newly formed nails. They make the nail lose its shine and transparency and may also be responsible for the friability of the nail. Nail keratin is also irregularly wavy, with shallow surface depressions corresponding, clinically, to the pits. The severity of the histological picture varies according to the clinical picture. The distal matrix and the nail bed are usually severely affected compared to the proximal matrix. Differential diagnosis with eczema is difficult, but the involvement of the periungual folds in the latter condition usually helps in performing the differential diagnosis [6,32,33,45]. Patients with trachyonychia have also been biopsied in the context of studying the inflammatory infiltrate affecting the proximal nail matrix, and the immunohistochemical characterization reported large numbers of Langerhans cells and intraepithelial T lymphocytes (with a reported CD4/CD8 ratio of 2:1). Only rare lymphocytes express the interleukin-2 receptor [6,27].

### 3.2. Clipping

The biopsy being an invasive procedure, especially in children, some authors investigated the diagnostic power of nail clippings in patients with AA [46,47]. The act of cutting the distal portion of a nail (3–4 mm) and processing the fragment for histopathologic analysis is a quick and painless procedure [48]. Onychomycosis should always be ruled out first with PAS staining. The nails of AA patients reveal, especially in the upper portion, cupoliform and shallow depressions, thin parallel slits giving rise to a flake-like appearance, and spongiosis. Subungual keratin is spared. The nail plate is usually thin, with architectural disorders of the corneocyte arrangement. This procedure, however, still needs standardization to be accepted as a routine procedure.

### 3.3. Imaging

The use of high-frequency ultrasonography (HFUS) on nail plates and nail beds for the evaluation of AA nails has also been reported [49]. HFUS is a non-invasive, in vivo technology that utilizes sound beams at frequencies typically above 15 MHz to produce detailed images of the superficial layers of the skin. Using a 33 Mhz transducer in B-mode (brightness mode: amplitude peaks seen as dots or pixels of varying brightness), loss of differentiation of the dorsal and ventral nail plates, onychorrhexis (tortuous hypoechoic lines between the layers of the nail plates), focal thickening of the nail plate layers, and koilonychia (inversion of the nail curvature forming a central depression) were found in a child with pitting and trachyonychia [49].

## 4. Management

No randomized controlled trials on the effect of treatments for AA nails are available, and no standardized evidence-based approaches exist. Data are solely based on small studies, case reports, and expert opinions. Most of the reported patients are supposed to have AA of the nails because they have AA of the scalp/body, but many of them have never been biopsied or reported isolated dystrophies also seen in AA but not diagnosed as such, especially trachyonychia. Usually, when AA of the scalp/body requires systemic treatment, nails are included in the treatment plan, but sometimes they may require topical/intralesional support. As stated, nail changes are often associated with severe forms of AA and represent a negative prognostic factor, so maybe their presence should always require a systemic treatment—nails should provide prognostic information that should always guide management decisions. When nail changes present as an isolated finding, instead, the best treatment should be selected according to the type and severity of clinical signs, number of affected nails, comorbidities, previous successful/unsuccessful treatments, patient age, and, above all, quality of life. It should be noted that data on nail changes are not usually collected properly in trials on AA or are not a primary endpoint, but they deserve a fair evaluation from baseline, possibly with an appropriate scoring system, because they may compromise quality of life or, being a negative prognostic factor, may compromise treatment outcome [50]. 

### 4.1. No Treatment

When not disfiguring, nail changes in AA can also not be treated but only followed up. In a 4- to 7-year follow-up of 23 children affected by AA and various nail abnormalities, most of them showed spontaneous recovery within a few years without treatment [10]. Another follow-up study of 12 patients with trachyonychia (including 2 patients with AA nails) showed that 50% of patients had total resolution or marked improvement of trachyonychia within 6 years, regardless of their treatment [51]. However, it is unknown if AA patients were part of this group. These data suggest that, especially for children, a wait-and-see approach may well be considered if the manual function is unaffected or there are no cosmetic concerns.

### 4.2. Cosmetic Treatment

Patients not undergoing medical treatment are always eligible for conservative approaches, including mild emollients for opaque trachyonychia and camouflage with nail polish for shiny trachyonychia. Occlusives, such as petrolatum or lanoline, and humectants, such as glycerin and propylene glycol, are usually the first options, but alpha-hydroxy acids and urea may also be added to increase the water-binding capacity of the fragile nail plate [52]. For opaque trachyonychia, nail polish is usually not helpful enough due to the very irregular surface [26]. A survey-based study reported regular manicure pedicures as a successful treatment of choice [12]. 

### 4.3. Topical Treatment

Clinicians are often reluctant to prescribe systemic treatments when the disease is restricted only to the nails, especially if the patient is a child or has comorbidities. In this case, topical treatment is probably the best option to start with, even if it may be time-consuming. Due to the slow growth rate of the nail plate and the difficulty with which the drug actives penetrate the nail tissues, it is, in fact, usually necessary to wait several months before seeing improvements. 

In a small case series reporting the treatment of trachyonychia, including two patients with AA of the nails, topical steroid treatment (mometasone furoate 0.1% ointment or betamethasone dipropionate 0.05% ointment) has been used for 6 months with good results, but not in all patients. Unfortunately, it is not reported if the two AA patients were among the improved ones [51]. In a study on 122 patients with nail dystrophies, 27 cases were associated with AA and presented with trachyonychia, pitting, and koilonychia, as well as ridging and fissuring. Seventeen patients were treated with topical steroids—clobetasol propionate 0.05% cream in particular—with a positive outcome [9]. Data on the use of topical steroids are limited, but it is safe to assume that they might work well as a first-line topical treatment option for many mild to moderately inflammatory nail conditions like psoriasis, lichen planus, and retronychia. However, it might be necessary to apply them for a long time to see results, and this may cause severe skin atrophy.

Another single case report reported the efficacy of tazarotene 0.1% gel once daily for a period of 3 months [53]. The rationale for treating such a disorder with retinoids may be linked to their actions in accelerating nail growth and normalizing keratinocyte differentiation [54,55]. Other treatments are reported for nail changes that occur in AA, but there is not always the same positive impact on nails and hair. In a study by Tosti et al. [6], for example, four patients experienced an improvement in nail changes after successful immunotherapy with squaric acid dibutylester started for hair loss. On the other hand, eight patients showed improvement of nail changes independently from the hair, and another eight patients had periodical worsening and improvement of nail changes unrelated to the course of AA of the hair. 

### 4.4. Intralesional Treatment

Inflammatory nail changes have been frequently treated with intralesional triamcinolone acetonide, especially when the matrix is affected. A couple of case reports and case series are also published for AA nails. 

An old study with four AA nail patients showed successful treatment with 5 mg/mL triamcinolone acetonide intralesional (matrix) injections at a 2- to 4-week interval for a total of three sessions. More severe cases subsequently received intermittent injections at 6- to 10-week intervals. Complete resolution was seen in three out of four patients, but relapses occurred in two out of three patients [56]. In this study, injections were performed with a jet injector—a device that should be, however, discouraged due to the potential risk of splashback of blood and the scarce manageability on a small and convex structure like the nail unit or a periungual thin skin of a child [57,58,59]. Intralesional injections should be performed with an insulin syringe with a built-in needle or a Luer lock syringe with a 30-gauge needle. The needle must be oblique to avoid injecting too close to the bone. It is also important to inject slowly until blanching from the fluid load is evident within the lunula area. Keeping the fluid at room temperature is also important to reduce pain during the injections. Grover et al. [60] reported improvement of nail dystrophies in over 70% of patients with 5 mg/mL triamcinolone acetonide intralesional (matrix) injections performed every month for 6 months. Among the 28 patients who completed the protocol, 4 had AA, but it is unclear if they were among the responders. Despite being a usually successful treatment, intralesional injections for inflammatory nail conditions still lack evidence regarding the optimal dosage, dilution, number and frequency of injections, and maximum duration of treatment. Injections are usually performed once a month, and if improvements are seen, they are continued until there is marked or complete improvement and then tapered for a few months. Koo et al. [61] proposed, with success, bimonthly intralesional (matrix) injections for pitted nails—a very good option when the disease is not very severe and affects children. Matrix injections are quite tolerable for the pediatric population, even without a digital block, especially if conducted on a few nails.

### 4.5. Systemic Treatment 

Systemic corticosteroids are also a potential treatment for AA nails, especially if manual function is impaired or there is a severe cosmetic disfigurement. A case report about severe onycholysis in a patient with AA universalis who received intramuscular triamcinolone acetonide 0.5 mg/kg/month for 3 months showed rapid improvement until restitutio ad integrum after 6 months and a mild recurrence after 1 year [62].

In a study on 122 patients with nail dystrophies, 27 cases were associated with AA and presented with trachyonychia, pitting, and koilonychia, as well as ridging and fissuring. Seventeen patients were treated with systemic steroids—in particular, intramuscular triamcinolone acetonide 40 mg/mL once a month for 5 months—because this treatment was judged more appropriate for the type and severity of associated AA of the hair. The outcome has been successful [9].

The literature reports no data on nails in patients with AA treated with methotrexate or other immunosuppressants like cyclosporin, mycophenolate mofetil, and azathioprine.

JAK inhibitors have become a relevant therapy option for patients with AA of the hair. Their effect on nails has not yet been investigated in depth. A couple of case reports showed the efficacy of tofacitinib, a JAK 1 and 3 inhibitor modifying the t-cell response to cytokines, for AA of the nails. Doses were reported from 10 to 15 mg/day. A 38-year-old patient with AA universalis showed functional recovery of AA-associated nail changes (trachyonychia and red lunulae) within 10 months with tofacitinib 10 mg/day [63]. Another case report about three patients with AA universalis and nail changes showed remission of these nail changes after treatment with tofacitinib at 10 mg/day or, when hair regrowth was not evident at this dosage, up to 15 mg/day [64]. Similarly, in a study with 15 AA patients with nail involvement, 11 showed improvement after a median of 5 months of tofacitinib 10 mg/day, followed by a decrease (5 mg/day) or increase (15 mg/day) of the dose according to the treatment response. Nail improvement occurred later than hair regrowth [21].

In another case report, tofacitinib was used at a dosage of 5 mg twice daily for 6 months and 2.5 mg twice daily for another 3 months, but the report was on unclassified trachyonychia because the histopathological assessment of the underlying inflammatory condition was denied by the parents of the 13-year-old patient [65].

Due to these observations, it seems that JAK inhibitors may be a suitable therapy option for both AA of the hair and nails in clinically relevant cases, but one should keep in mind that, until now, no reports about the use of JAK inhibitors other than tofacitinib are available for nail dystrophies. Since baricitinib and ritlecitinib were recently approved for AA by the FDA/EMA [66,67], we should expect reports on their effects on nails in the near future. As stated previously, the nail matrix, like the hair follicle, may exhibit gene expression similar to hair follicle keratinocytes in AA. In this case, JAK inhibitors would be expected to reverse nail dystrophy as well as hair loss in affected patients.

## 5. Conclusions

AA of the nails can precede or follow AA of the hair by years, but it can also appear as an isolated finding, never being associated with hair loss. In most cases, they develop simultaneously, especially in severe forms of AA affecting children. Many adult patients with severe forms of AA do not present nail changes. The reported frequency of nail abnormalities is, in fact, inferior compared to hair loss. Geometrical grid-like pitting, opaque trachyonychia, and leukonychia are the most often reported changes. Checking the nails in patients with AA or suspecting this diagnosis in patients presenting with isolated findings compatible with this diagnosis is important because, despite the fact the disease has no scarring potential and is not usually disfiguring, it could stress the patient, limit daily activities and reduce productivity impacting on costs. The clinical picture is, unfortunately, not specific, and a biopsy should be performed to reach a definitive diagnosis. Due to the favorable prognosis, this procedure is, however, rarely performed. It would be desirable to have data on clipping standardized and accepted as a routine procedure. This would be extremely valuable, especially for pediatric patients. A longitudinal biopsy should be, however, necessary to further explore the pathophysiology of AA nail changes, which is useful not only for a more accurate diagnosis and treatment plans but also for the development of new therapeutic targets. A better investigation of nail pathology can also help to better elucidate the inflammatory pathways and cytokine profiles involved. Nail changes are, however, rarer compared to hair loss and mostly affect children—not the ideal population to perform such clinical studies.

Overall, nail abnormalities associated with AA are not disfiguring and often respond well to therapy for hair loss. This may be an explanation for why the literature on this topic is scarce. A consensus for the best treatment approach according to age and severity is, however, very much needed. As for AA of the hair, JAK inhibitors could be the most promising treatment option in the near future, and the new topical formulas (ruxolitinib and tofacitinib) may even prove superior from a safety point of view, especially in children, old patients, and those with comorbidities that do not allow systemic JAK inhibitor prescription. 

## Figures and Tables

**Table 1:** A summary of AA nail changes, according to their documented nail alterations as well as their frequency in each study. Yellow: study included children. Orange: study included children and adults. Red: study included adults. White: no data on the age of the study population are available. The second row shows the general nail changes of AA patients by study. The following rows show specific nail changes as a percent of all reported nail changes in the study population. The last column shows the combined percentage of nail changes from several studies.

Nail Changes			Tosti 1991 *n* = 1095 [6]	Tosti 1994 *n* = 272 [10]	Sharma 1996 *n* = 201 [14]	Gordon 1996 *n* = 48 [15]	Sharma 1998 *n* = 1000 [7]	Sharma 1998 *n* = 761 [7]	Sharma 1998 *n* = 239 [7]	Tan 2002 *n* = 219 [16]	Ghandi 2003 *n* = 100 [17]	Goh 2006 *n* = 513 [18]	Kasumagic 2009 *n* = 200 [8]	Al-Mutairi 2011 *n* = 2962 [19]	Cho 2012 *n* = 287 [4]	Ranawaka 2014 *n* = 290 [20]	Roest 2018 *n* = 256 [12]	Lee 2018 *n* = 33 [21]	Starace 2020 *n* = 27 [9]	Shakoei 2024 *n* = 197 [22]	Overall *n* = 7476
all			25.6% (280/1095)	46.3% (126/272)	29.9% (60/201)	66.7% (32/48)	19.8% (198/1000)	16.8% (128/761)	29.3% (70/239)	10.5% (23/219)	44% (44/100)	38% (195/513)	24.5% (49/200)	10.0% (297/2962)	7.3% (21/287)	9.3% (27/290)	64.1% (164/256)	45.5% (15/33)		83.8% (165/197)	22.1% (1696/7673)
	pitting		85.7% (240/280)	73% (92/126)	65% (39/60)		70.2% (139/198)	73.4% (94/128)	64.3% (45/70)		63.6% (28/44)		79.6% (39/49)				46.3% (76/164)	93.3% (14/15)	92.6% (25/27)	63.6% (105/165)	70.7% (797/1128)
		mild pitting		44.4% (56/126)																	44.4% (56/126)
		severe pitting/ crumbling		28.6% (36/126)													41.5% (68/164)				35.9% (104/290)
	trachyonychia		14.3% (40/280)	25.4% (32/126)	13.3% (8/60)		41.9% (83/198)	48.4% (62/128)	30% (21/70)		20.5% (9/44)		14.3% (7/49)				48% (46/164)	6.7% (1/15)	63% (17/27)	17.6% (29/165)	24.1% (272/1128)
		rough/opaque trachonychia	3.2% (9/280)	5.6% (7/126)			10.6% (21/198)														6.1% (37/604)
		shiny trachyonychia	11.1% (31/280)	19.8% (25/126)	3.3% (2/60)															1.2% (2/165)	9.5% (60/631)
	longitudinal ridging				13.3% (8/60)		39.4% (78/198)	38.3% (49/128)	15.7% (11/70)		22.7% (10/44)						14% (23/164)		77.8% (21/27)	55.8% (92/165)	35.3% (232/658)
	koilonychia																4.9% (8/164)		55.6% (15/27)	6.7% (11/165)	9.6% (34/356)
	onchomadesis Beau’s lines			2.4% (3/126)							4.5% (2/44)						1.8% (1+2/164)	13.3% (2/15)		1.8% (3/165)	2.5% (13/514)
	onychorrhexis distal notching onchoschisis brittle nails						5% (10/198)				4.5% (2/44)		6.1% (3/49)				16.5% (8+9+10/164)			38.2% (53+10/165)	16.9% (105/620)
	leukonychia			0.8% (1/126)	1.7% (1/60)		13.6% (27/198)										38.4% (63/164)			21.2% (35/165)	17.8% (127/713)
	pigmentation discoloration				3.3% (2/60)															3.0% (5/165)	3.1% (7/225)
	red (mottled) lunula			4.8% (6/126)			0% (0/198)										7.9% (13/164)	13.3% (2/15)		1.2% (2/165)	3.4% (23/668)
	disappearing lunula																0.6% (1/164)			8.5% (14/165)	4.6% (15/329)
	onycholysis																1.8% (3/164)	6.7% (1/15)		3.0% (5/165)	2.6% (9/344)
	ragged cuticles										4.5% (2/44)									4.8% (8/165)	4.8% (10/209)
	slow growth																0.6% (1/164)			4.2% (7/165)	2.4% (8/329)
